# Inhibitory Effect of Dried Pomegranate Concentration Powder on Melanogenesis in B16F10 Melanoma Cells; Involvement of p38 and PKA Signaling Pathways

**DOI:** 10.3390/ijms161024219

**Published:** 2015-10-13

**Authors:** Su Jin Kang, Beom Rak Choi, Eun Kyoung Lee, Seung Hee Kim, Hae Yeon Yi, Hye Rim Park, Chang Hyun Song, Young Joon Lee, Sae Kwang Ku

**Affiliations:** 1 The Medical Research Center for Globalization of Herbal Medicine, Daegu Haany University, Gyeongsan 712-715, Korea; E-Mails: vegonia1@hanmail.net (S.J.K.); kkong0305@hanmail.net (E.K.L.); dvmsong@hotmail.com (C.H.S.); 2 Department of Preventive Medicine, College of Korean Medicine, Deagu Haany University, Gyeongsan 712-715, Korea; 3 Research Institute, Health-Love Co., Ltd., Anyang 431-060, Korea; E-Mails: brchoi@health-love.com (B.R.C.); key7413@health-love.com (S.H.K.); Leehaeyun@health-love.com (H.Y.Y.); hrpark@health-love.com (H.R.P.); 4 Department of Histology and Anatomy, College of Korean Medicine, Daegu Haany University, Gyeongsan 712-715, Korea

**Keywords:** pomegranate concentrate powder, whitening, anti-melanin, p38

## Abstract

Plants rich in antioxidant substances may be useful for preventing skin aging. Pomegranates, containing flavonoids and other polyphenolic compounds, are widely consumed due to their beneficial properties. We examined the underlying mechanisms of dried pomegranate concentrate powder (PCP) on melanin synthesis in B16F10 melanoma cells. The antioxidant effects of PCP were determined by measuring free radical scavenging capacity and transcript levels of antioxidant enzymes. To explore the inhibitory effects of PCP on melanin synthesis, we measured tyrosinase activity and melanin content in α-melanocyte stimulating hormone (α-MSH)-stimulated B16F10 cells. In addition, the levels of tyrosinase-related protein-1 (TRP-1), TRP-2, tyrosinase, and microphthalmia-associated transcription factor (MITF) expression were determined by Western blotting. Changes in the phosphorylation status of protein kinase A (PKA), cAMP response element-binding protein (CREB), mitogen-activated protein kinases (MAPKs), phosphatidylinositol 3-kinase (PI3K), serine/threonine kinase Akt, and glycogen kinase 3β (GSK3β) were also examined. The free radical scavenging activity of PCP increased in a dose-dependent manner. In PCP-treated B16F10 cells, transcript levels of glutathione peroxidase-1 (GPx-1) were increased compared with α-MSH-stimulated cells. In addition, PCP led to the down-regulation of phospho-p38, phospho-PKA, phospho-CREB, phospho-GSK3β, MITF, and TRP-1 compared with α-MSH-stimulated B16F10 cells. We believe this effect may be associated with PCP activity, which leads to the inhibition of melanin production and tyrosinase activity. These results suggest that PCP decreases tyrosinase activity and melanin production via inactivation of the p38 and PKA signaling pathways, and subsequently decreases phosphorylation of CREB, MITF, and melanogenic enzymes. These observations provided new insights on the molecular mechanisms of the skin-whitening property of PCP.

## 1. Introduction

Melanin, a pigment produced by epidermal melanocytes, is the main determinant of skin color and contributes to protection against ultraviolet (UV) irradiation [1]. Melanogenesis is a complex process involving tyrosinase and tyrosinase-related proteins (TRPs). Tyrosinase plays an important role in regulating melanin formation, in which l-tyrosine is hydroxylated to l-dihydroxyphenylalanine (l-DOPA), and l-DOPA is oxidized into the corresponding *o*-quinone [2]. In addition, tyrosinase and TRPs are controlled by microphthalmia-associated transcription factor (MITF) in melanocytes. Moreover, it was recently reported that hyperpigmentation is prevented by the inhibition of melanogenesis [3,4,5].

Melanin synthesis is regulated by the transduction of several signals that play important functions in the process of melanogenesis. UV radiation and α-melanocyte stimulating hormone (α-MSH) activate adenyl cyclase to increase cAMP, which triggers the activation of cAMP-dependent protein kinase A (PKA) and several regulatory proteins [6]. Thereafter, PKA leads to the phosphorylation of cAMP response element binding protein (CREB), and the phosphorylation of CREB has been found to activate MITF transcription [6,7]. Mitogen-activated protein kinases (MAPKs), p38, extracellular response kinase (ERK), and c-Jun N-terminal (JNK) are important factors in melanogenesis. Recent studies have reported that p38 MAPK is involved in MITF regulation [8]. In addition, activation of the p38 MAPK signaling pathway increases the transcription of tyrosinase, which activates melanin synthesis [9,10].

In the process of melanogenesis, hydrogen peroxide (H_2_O_2_) and other reactive oxygen species (ROS) accumulate, leading to oxidative stress-induced damage of melanocytes [11]. ROS damage cellular lipids, proteins and DNA. Furthermore, ROS can inhibit the formation of collagen, disrupt cellular renewal cycles and cause imbalances between ROS and antioxidant systems [12,13,14]. Thus, ROS scavengers and inhibitors of ROS production may suppress the melanogenesis pathway and protect against skin damage.

Plant-derived natural antioxidants are a focus of skin aging prevention due to their potential to scavenge ROS and inhibit the UV-induced signal transduction pathway [15]. Therefore, antioxidant substances represent a promising strategy for reducing melanogenesis and skin aging. Pomegranates are rich in ellagic acid and polyphenols, which include flavonoids and hydrolyzable tannins [16,17,18]. Polyphenolics, contained in several plants, form the important part of the diet and exerts effective free radical scavenging and antioxidant activities. Studies have demonstrated that pomegranates exert strong anti-oxidant effects due to their free radical scavenging capacity and antioxidant properties [19,20]. Recently, it was demonstrated that the skin-whitening effects of pomegranates are due to the inhibition of melanocyte proliferation and melanin synthesis by tyrosinase in melanocytes [18,21,22]. However, the inhibitory effects of pomegranates on melanogenesis have not been fully examined. Pomegranate contains large amounts of ellagic acid and other organic materials, including flavonoids and polyphenols, more than either red wine and polyphenols [16,18,23]. These characteristics provide protection against heart disease and cancer [24,25,26,27,28,29]. In addition, pomegranate possesses anti-proliferative [30], anti-inflammatory [31], and anti-tumorigenic functions [32].

This study investigated the inhibitory effects of dried pomegranate concentrate powder (PCP) on melanogenesis in B16F10 melanoma cells and evaluated the potential antioxidant characteristics of PCP. In addition, the underlying mechanisms of the effects of PCP on melanin synthesis were explored in B16F10 melanoma cells by determining free radical scavenging capacity, tyrosinase activity, and melanin content. Moreover, the expression of TRP-1, TRP-2, and tyrosinase was examined, along with the MAPKs and PKA/CREB pathways. Finally, the PI3K/Akt signaling pathway was investigated in B16F10 cells. The murine B16F10 cell line was selected as a model of human melanoma since it produces melanin in response to activation by α-MSH [33,34].

## 2. Results

### 2.1. Cytotoxicity of PCP in B16F10 Cells

**Figure 1:**
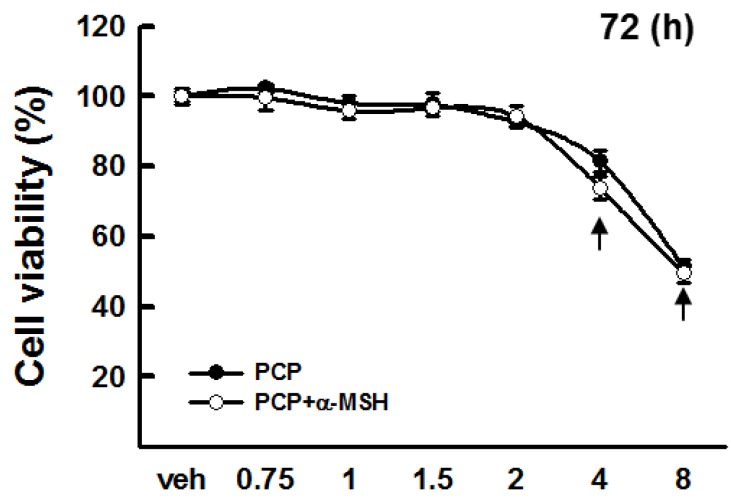
Cytotoxicity of pomegranate concentrate powder (PCP) in murine B16F10 melanoma cells. B16F10 cells were treated with various concentrations of PCP (0.75, 1, 1.5, 2, 4, and 8 mg/mL) in the absence or presence of α-MSH for 72 h. Values are expressed as the means ± SD from three independent experiments; PCP, pomegranate concentrate powder; α-MSH, α-melanocyte-stimulating hormone; and veh, vehicle control. Arrow indicates a significant (*p* < 0.001) decrease in cell viability compared with the control (veh) using the LSD test.

We first evaluated the cytotoxic effects of PCP on B16F10 cells using the MTT assay. PCP (0.75, 1, 1.5, 2, 4, and 8 mg/mL) was added to B16F10 cells in the absence or presence of α-MSH. As shown in Figure 1, no significant differences were observed in the viability of B16F10 cells treated with PCP concentrations ranging from 0.5 to 2 mg/mL *versus* untreated control cells (Figure 1). Thus, we used PCP concentrations of 0.75, 1, and 1.5 mg/mL for subsequent experiments in B16F10 cells.

### 2.2. Free Radical Scavenging Activity of PCP

**Figure 2:**
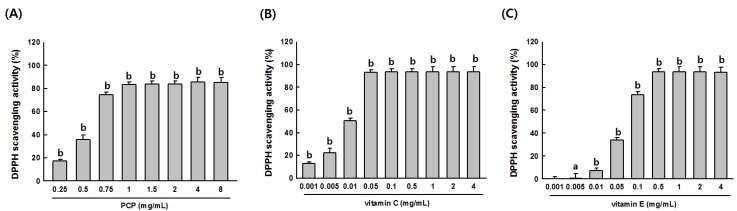
Antioxidant characteristics of PCP. DPPH scavenging activity was examined at (**A**) PCP concentrations of 0.75, 1, 1.5, 2, 4, and 8 mg/mL; (**B**) vitamin C concentrations of 0.01, 0.05, 0.1, 0.5, 1, 2, and 4 mg/mL; and (**C**) vitamin E concentrations of 0.01, 0.05, 0.1, 0.5, 1, 2, and 4 mg/mL. Values are expressed as the means ± SD from three independent experiments. PCP, pomegranate concentrate powder; Vit C, vitamin C; Vit E, vitamin E; DPPH, 1-diphenyl-2-picrylhydrazyl radical; 2,2-diphenyl-1-(2,4,6-trinitrophenyl)hydrazyl; ABTS, 2,3-azinobis (3-ethyl-benzothiazoline-6-sulfonic acid. ^a^ *p* < 0.05 or ^b^ *p* < 0.001 compared with the control (veh) using the Mann-Whitney *U*-test.

Significant increases in DPPH radical scavenging activities were detected in samples treated with vitamin A (1 mg/mL), vitamin C (1 mg/mL), and PCP at concentrations ranging from 0.25 to 8 mg/mL. Vitamin A, vitamin C, and PCP significantly increased the radical scavenging activity in a concentration-dependent manner. Notably, PCP at concentrations 1 mg/mL or higher exhibited scavenging activity similar to those of the positive controls (vitamin C- and vitamin E-treated samples) (Figure 2A–C). The ABTS assay was also performed to confirm the antioxidant property of PCP. Significant increases in ABTS radical scavenging activities were observed at PCP concentrations ranging from 0.25 to 8 mg/mL. Calculated IC_50_ values for DPPH and ABTS activity with PCP (0.25 to 8 mg/mL) were 0.52 and 0.54 mg/mL, respectively.

### 2.3. Tyrosinase Activity of PCP

**Figure 3:**
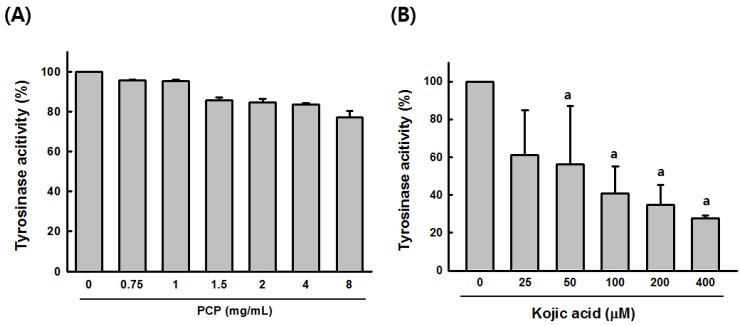
Inhibitory effects of PCP on melanogenesis. (**A**) The effect of PCP on mushroom tyrosinase activity was determined at concentrations of 0.75, 1, 1.5, 2, 4, and 8 mg/mL; (**B**) the effect of Kojic acid on mushroom tyrosinase activity was observed at concentrations of 25, 50, 100, 200, and 400 μM. Values are expressed as the means ± SD from three independent experiments. PCP, pomegranate concentrate powder. ^a^ *p* < 0.05 compared with the control (veh) using the LSD test.

To examine the tyrosinase effect, l-DOPA oxidation with mushroom-tyrosinases was determined at 0.75, 1, 1.5, 2, 4, and 8 mg/mL PCP, respectively. At 4 and 8 mg/mL, PCP slightly reduced mushroom tyrosinase activity to 14.33% ± 1.39% and 23.98% ± 3.316%, respectively. Kojic acid significantly inhibited mushroom tyrosinase activity in a concentration-dependent manner (Figure 3).

### 2.4. Intracellular Tyrosinase Activity of PCP in B16F10 Cells

**Figure 4:**
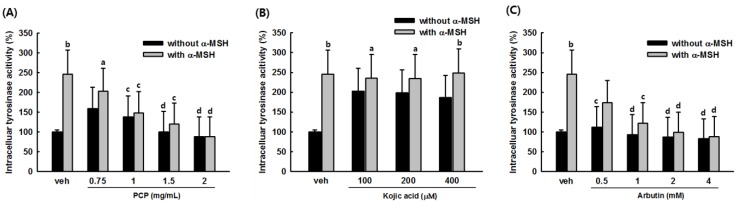
Effects of PCP on tyrosinase activity in B16F10 cells. (**A**) Tyrosinase activity was determined in B16F10 cells in the absence or presence of α-MSH (100 nM). B16F10 cells were exposed to various concentrations of PCP (0.75, 1 or 1.5 mg/mL) for 72 h after treatment with or without α-MSH for 24 h; (**B**) Kojic acid (50, 100, 200, and 400 μM) was applied for 72 h following treatment with or without α-MSH for 24 h; (**C**) Arbutin (0.5, 1, 2, and 4 mM) was applied for 72 h following treatment with or without α-MSH for 24 h. Values are expressed as the means ± SD from three independent experiments. PCP, pomegranate concentrate powder; α-MSH, α-melanocyte-stimulating hormone. ^a^ *p* < 0.05 or ^b^ *p* < 0.01 compared with the control (veh) using the LSD test; ^c^ *p* < 0.05 or ^d^ *p* < 0.01 compared with α-MSH treatment only using the LSD test.

To clarify the tyrosinase inhibitory effect of PCP on melanogenesis, we determined the intracellular tyrosinase activity of PCP-treated B16F10 melanoma cells with or without α-MSH stimulation. As shown in Figure 4A, an approximately 2.2-fold increase in cellular tyrosinase activity was observed in α-MSH-stimulated cells compared with unstimulated cells. Tyrosinase activity of 0.75, 1 and 1.5 mg/mL PCP-treated cells was reduced by 16.5%, 37.7%, and 48.6%, respectively, compared with α-MSH-stimulated cells (Figure 4A). Kojic acid at 50, 100, 200, and 400 μM also decreased intracellular tyrosinase activity by 27.8%, 48.1%, 57.1%, and 61.1%, respectively, compared with α-MSH-stimulated cells (Figure 4B). Arbutin at 0.5, 1, 2, 4 μM also decreased intracellular tyrosinase activity by 29.17%, 50.44%, 59.83%, and 64.03%, respectively, compared with α-MSH-stimulated cells. Arbutin works by inhibiting the enzyme tyrosinase, a key enzyme in the synthesis of melanin.

### 2.5. The Effects of PCP on Anti-Melanin Formation in B16F10 Cells

**Figure 5:**
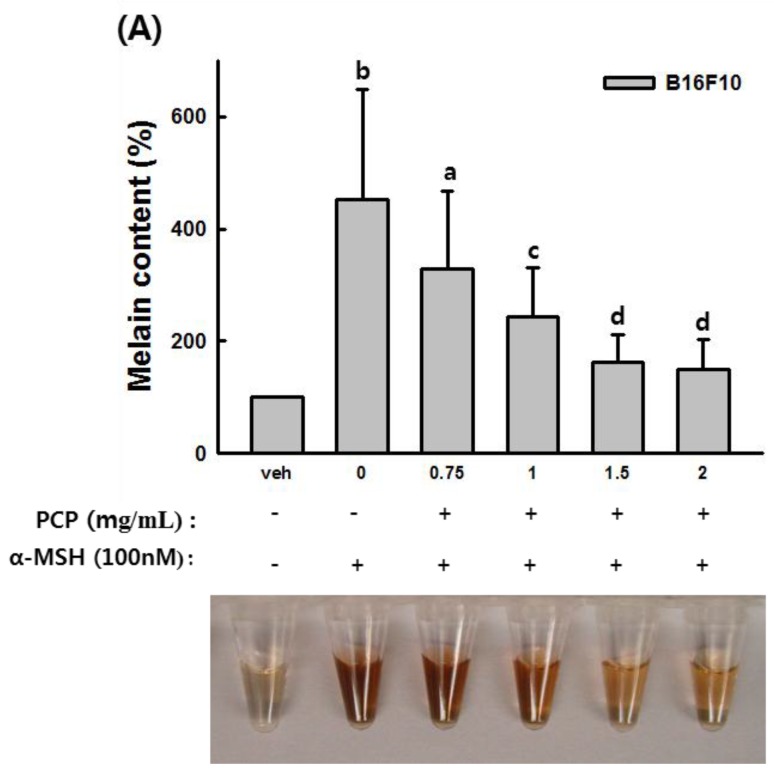

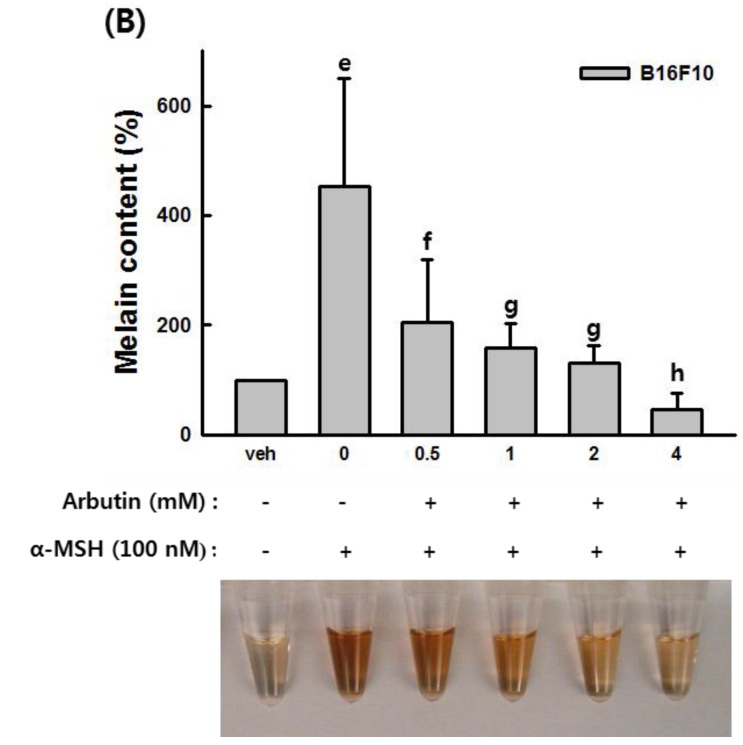
Anti-melanin formation effect of PCP against α-MSH stimulation in B16F10 cells. (**A**) Melanin production was determined in B16F10 cells in the absence or presence of α-MSH (100 nM). B16F10 cells were exposed to various concentrations of PCP (0.75, 1 or 1.5 mg/mL) for 72 h following treatment with or without α-MSH for 24 h; (**B**) Arbutin (0.5, 1, 2, and 4 mM) was applied for 72 h following treatment with or without α-MSH for 24 h. Values are expressed as the means ± SD from three independent experiments. PCP, pomegranate concentrate powder; α-MSH, α-melanocyte-stimulating hormone. ^a^ *p* < 0.05 or ^b^ *p* < 0.01 compared with the control (veh) using the LSD test; ^c^ *p* < 0.05 or ^d^ *p* < 0.01 compared with α-MSH treatment only using the LSD test; ^e^ *p* < 0.05 or ^f^ *p* < 0.05 compared with the control (veh) using the Mann-Whitney *U*-test; ^g^ *p* < 0.01 or ^h^ *p* < 0.001 compared with α-MSH treatment only using the Mann-Whitney *U*-test.

To confirm the effect of PCP on melanin production, we performed a melanin content assay. Results are shown in Figure 5. Melanin content of α-MSH-stimulated cells increased approximately 3.5-fold relative to vehicle-treated control cells. In contrast, 0.75, 1, 1.5, and 2 mg/mL PCP significantly decreased melanin production by 27.1%, 46.0%, 64.2%, and 66.8%, respectively, compared with α-MSH-stimulated B16F10 cells. Arbutin at 0.5, 1, 2, and 4 mM also reduced melanin formation by 54.5%, 64.7%, 70.8%, and 89.6%, respectively, compared with α-MSH-stimulated cells (Figure 5).

### 2.6. The Effects of PCP on Tyrosinase, TRP-1, TRP-2, and MITF in B16F10 Cells

**Figure 6:**
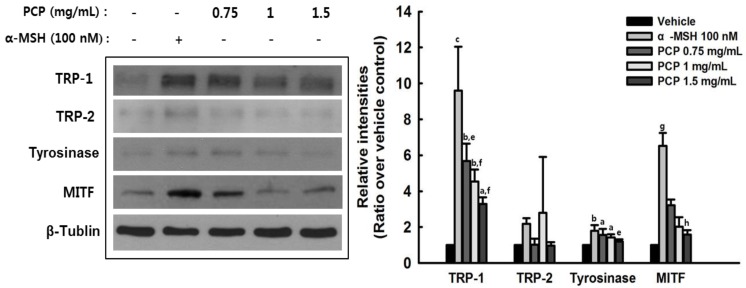
Effects of PCP on melanogenesis-related protein expression. B16F10 cells were exposed to various concentrations of PCP (0.75, 1 or 1.5 mg/mL) for 48 h following treatment with or without α-MSH (100 nM) for 24 h. Cellular proteins levels of TRP-1, TRP-2, tyrosinase, and MITF were examined by Western blot analysis. PCP, pomegranate concentrate powder; α-MSH, α-melanocyte-stimulating hormone; TRP-1, tyrosinase related protein 1; TRP-2, tyrosinase related protein 2; MITF, microphthalmia-associated transcription factor. ^a^ *p* < 0.05; ^b^ *p* < 0.01 or ^c^ *p* < 0.001 compared with the control (veh) using the LSD test; ^d^ *p* < 0.05; ^e^ *p* < 0.01 or ^f^ *p* < 0.001 compared with α-MSH treatment only using the LSD test; ^g^ *p* < 0.05 compared with the control (veh) using the Mann-Whitney *U*-test; ^h^ *p* < 0.05 compared with α-MSH treatment only using the Mann-Whitney *U*-test.

Since inhibitors of hypopigmentation regulate melanin production via suppression of cellular tyrosinase activity, protein levels of tyrosinase, TRP-1, TRP-2, and MITF were examined in PCP-treated B16F10 cells with or without α-MSH. In the absence α-MSH, PCP treatment did not stimulate the expression levels of TRP-1, tyrosinase, and MITF and pigment synthesis (data not shown). α-MSH pretreatment increased the levels of TRP-1, tyrosinase, and MITF, while PCP with α-MSH pretreatment decreased the expression levels of TRP-1, tyrosinase, and MITF (Figure 6).

### 2.7. The Effects of PCP on p-JNK, p-p38, p-ERK, p-PKA, p-CREB, and p-GSK3β Protein Levels

**Figure 7:**
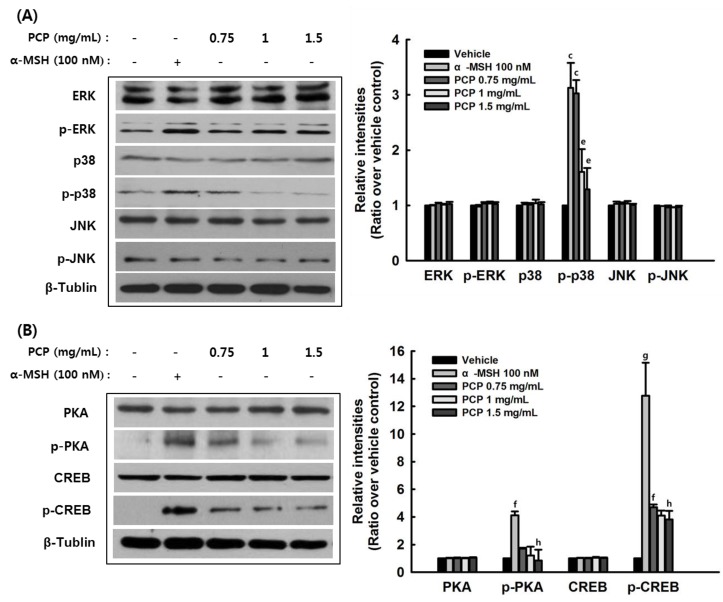

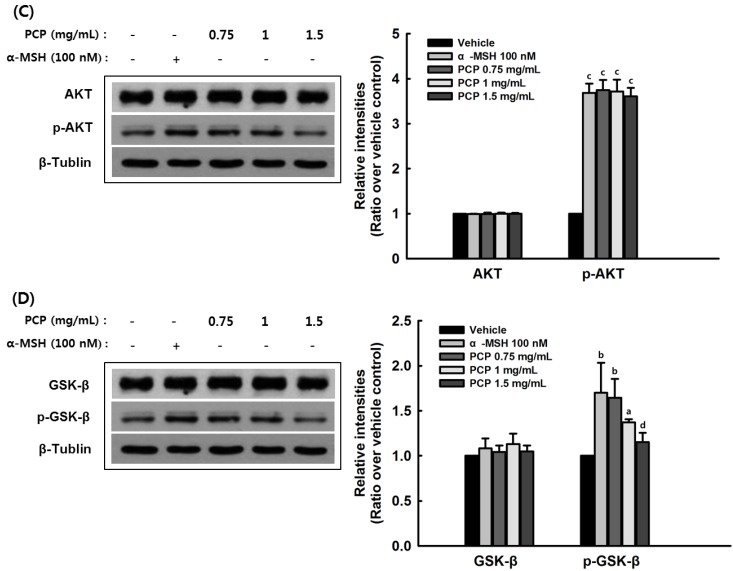
Effects of PCP on melanogenesis-related signaling pathways. B16F10 cells were exposed to various concentrations of PCP (0.75, 1 or 1.5 mg/mL) for 48 h following treatment with or without α-MSH (100 nM) for 24 h. (**A**) ERK, p-ERK, 38 MAPK, p-38, JNK, p-JNK; (**B**) PKA, p-PKA, CREB, p-CREB; (**C**) AKT; p-AKT (**D**) GSK-β, p-GSK-β, and β-tubulin protein levels were examined by Western blot analysis. PCP, pomegranate concentrate powder; α-MSH, α-melanocyte-stimulating hormone; p-ERK, phosphorylated extracellular signal-regulated kinase; p-p38, phosphorylated p38 mitogen-activated protein kinase; p-JNK, phosphorylated c-Jun N-terminal kinase; p-PKA, phosphorylated c-AMP-dependent kinase A; p-CREB, cAMP response element-binding protein; glycogen kinase 3β, GSK3β. ^a^ *p* < 0.05; ^b^ *p* < 0.01 or ^c^ *p* < 0.001 compared with the control (veh) using the LSD test; ^d^ *p* < 0.01 or ^e^ *p* < 0.001 compared with α-MSH treatment only using the LSD test; ^f^ *p* < 0.05 or ^g^ *p* < 0.01 compared with the control (veh) using the Mann-Whitney *U*-test; ^h^ *p* < 0.05 compared with α-MSH treatment only using the Mann-Whitney *U*-test.

Various signaling pathways regulate melanin pigment formation. Melanogenic gene expression is regulated primarily by MITF, as well as PKA, CERB, MAPKs, PI3K, AKT, and GSK3β. Since MAPKs play pivotal roles in the regulation of melanogenesis, we examined protein levels of p-PKA, p-CREB, p-JNK, p-p38, p-ERK, p-PI3K, p-AKT, and p-GSK3β in α-MSH-stimulated B16F10 cells to further demonstrate the effects of PCP on melanogenesis. In the absence α-MSH, PCP treatment did not stimulate the expression levels of p-JNK, p-38, p-ERK, p-PKA, p-CREB, and p-GSK3β (data not shown). In addition, α-MSH treatment increased the levels of p-38, p-PKA, and p-CREB, while PCP with α-MSH pretreatment decreased the expression levels of p-38, p-PKA, and p-CREB (Figure 7).

### 2.8. Suppression of α-MSH-Induced Alterations through p38 Inhibition

**Figure 8:**
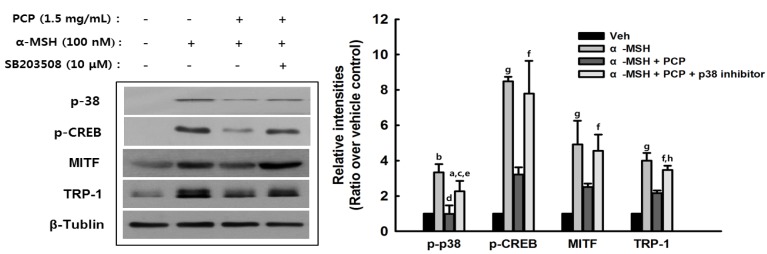
B16F10 cells were incubated with 10 μM SB203508 for 1 h before 1.5 mg/mL PCP for 48 h following treatment with or without α-MSH (100 nM) for 24 h. 38 MAPK, p-38, p-CREB, MITF, TRP-1, and β-tublin examined by Western blot analysis. PCP = pomegranate concentrate powder, α-MSH = α-melanocyte-stimulating hormone, p-p38 = phosphorylated p38 mitogen-activated protein kinase, p-PKA = phosphorylated c-AMP-dependent kinase A, p-CREB = cAMP response element-binding protein, TRP-1 = tyrosinase related protein 1, MITF = microphthalmia-associated transcription factor. ^a^ *p* < 0.01 or ^b^ *p* < 0.001 compared with the control (veh) using the LSD test; ^c^ *p* < 0.05 or ^d^ *p* < 0.001 compared with α-MSH treatment only using the LSD test; ^e^ *p* < 0.01 compared with α-MSH + PCP treatment using the LSD test; ^f^ *p* < 0.05 or ^g^ *p* < 0.01 compared with the control (veh) using the Mann-Whitney *U*-test; ^h^ *p* < 0.01 compared with α-MSH + PCP treatment using the Mann-Whitney *U*-test.

To confirm that the MPKA signaling pathways are related to the regulation of PCP on melanogenesis in MSH-stimulated cells, p38, JNK and ERK inhibitors (SB203508, SP600125 and PD98059) were added to test the melanogenesis-altering effects of PCP. As shown in Figure 8, treatment with SB203508 and PCP suppressed the levels of p-38 and TRP-1. In contrast, JNK and ERK inhibitors did not affect α-MSH-induced alterations (data not shown).

### 2.9. Changes in the Transcript Levels of SOD, CAT, GPx-1, and MITF after PCP Treatment in B16F10 Cells

**Figure 9:**
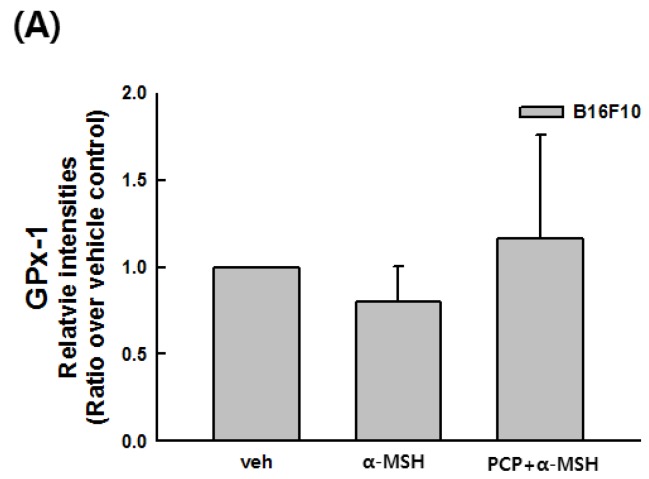

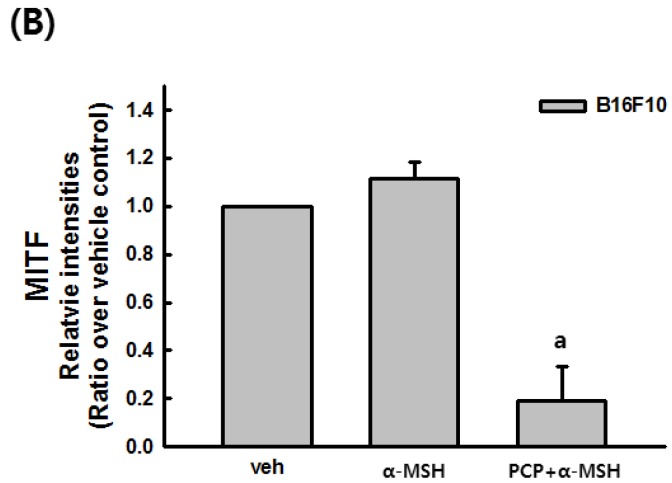
Transcript levels of (**A**) GPx-1 and (**B**) MITF were measured by real-time PCR. B16F10 cells were exposed to PCP (1.5 mg/mL) for 48 h following treatment with or without α-MSH for 24 h. Values are expressed as the means ± SD from three independent experiments. PCP, pomegranate concentrate powder; α-MSH, α-melanocyte-stimulating hormone; veh, control vehicle; GPx-1, glutathione peroxidase 1; MITF, microphthalmia-associated transcription factors. ^a^ *p* < 0.05 compared with α-MSH treatment only using the LSD test.

Transcript levels of SOD, CAT, GSR, GPx-1, and MITF were assessed in PCP-treated cells with or without α-MSH by real-time PCR. As shown in Figure 9, α-MSH stimulation decreased transcript levels of GPx-1 to counter oxidative stress in B16F10 cells. In contrast, PCP treatment restored the levels of GPx-1. At a concentration of 1.5 mg/mL PCP, GPx-1 transcript levels were increased to similar levels as untreated cells (Figure 9A). α-MSH stimulation decreased the expression of CAT and SOD; however, PCP did not affect those of CAT or SOD (data not shown). In addition, PCP treatment reduced the α-MSH-induced increase in MITF in B16F10 cells (Figure 9B).

## 3. Discussion

A wide variety of polyphenolic compounds derived from natural products possess potent antioxidant and anti-melanogenic activities [19,20,21,22]. Thus, this study examined the effects of PCP on the melanogenesis signaling pathway activated by α-MSH in B16F10 cells. α-MSH was used as a cAMP inducer to activate melanin synthesis [33]. Evidence has shown that α-MSH activates the cAMP signaling pathway and regulates melanocyte pigmentation [35].

We determined that the evaluated concentrations of PCP (0.75, 1, 1.5, and 2 mg/mL) exhibited no cytotoxicity in B16F10 cells. Thus, these PCP concentrations were used for subsequent experiments.

The potential antioxidant effects of PCP were first evaluated. PCP used in this study contains ellagic acid (0.36 mg/g) and punicalagin A and B (0.07 mg/g). Ellagic acid is a naturally-occurring polyphenolic compound found in many natural sources. It has been demonstrated to show anticarcinogenic, antifibrotic, and antioxidantative effects. In addition, it has been reported that ellagic acid inhibited UV-induced skin pigmentation of guinea pigs [18] and human skin [36]. Punicalagins are often noted as punicalagin; however, it is found naturally as two reversible α- and β-anomers. The punicalagins show a potent bioactivity to hydrolyze into ellagic acid and across the mitochondrial membrane *in vitro*. Punicalagin have a protection effect against oxidative damages to lipids, amino acids, and guanosine [37,38]. HPLC analysis showed that the total phenolic acid content of PCP was 0.43 μg/mg. To more understand the effects of PCP, further studies are required to analyze and examine the active components of PCP including ellagic acid and punicalagins A and B. PCP showed strong scavenging activity (IC_50_ value of the phenolic acids was 0.83 μg/mL in this study) compared with the DPPH results of Gismondi *et al.* [39]; *Eremomastax speciosa* (Hochst.) Cufod., and *Aframomum melegueta* K. Schum. (AM) had IC_50_ values of 0.36 and 0.55 μg extract per mL, respectively. Our data indicate that PCP exerts strong DPPH and ABTS radical scavenging effects. In addition, PCP increased the transcript level of GPx-1, which may be involved in the inhibition of oxidative damage in B16F10 cells. These results suggest that the effects of PCP on melanogenesis are related to the regulation of oxidative stress. Our study did not evaluate the secondary metabolites of PCP; thus, the amount of ROS in cells or the oxidative damage was not clarified. Gismondi *et al.* [40] showed that secondary metabolites affects antioxidant protein of cells. In this point, further study is required to examine the effects of the antioxidant protein in B16F10 cells of the secondary metabolites of pomegranate. Pomegranate seeds and peel exert the strongest antioxidant effects, based on corrected literature data. Peels have greater activity than seeds [41,42]; this difference in antioxidant activity may arise from differences in their phenolic compositions. In addition, an aqueous extract of dried whole peel had the highest antioxidant activity compared with methanolic pomegranate peel extracts [42]. Melanogenesis is an oxidizing process; more specifically, melanogenesis-induced reduction of glutathione and cysteine is an oxidative stress response in melanocytes. When ROS levels exceed endogenous antioxidant capacity, oxidative damage results, which may contribute to the progression of skin aging [43,44]. Thus, many antioxidants are used to inhibit melanin production by counteracting the damaging effects of ROS. Antioxidant enzymes, including GPx-1, SOD, and CAT, are important factors in maintaining redox homeostasis [45,46]. In addition, the amount of enzymatic antioxidants such as CAT and GPx was changed in B16F10 cells after essential oil and propolis [40]. GPx-1 is a ubiquitously expressed cytosolic enzyme with peroxidase activity whose main biological role is to protect the cell from oxidative damage, and is a pivotal enzyme responsible for the removal of H_2_O_2_ [47]. In the process of melanogenesis, H_2_O_2_ is overproduced and leads to oxidative stress in skin cells [48].

To determine whether PCP inhibits the process of melanin biosynthesis, tyrosinase activity, and melanin production were examined in B16F10 cells in the absence or presence of α-MSH. Protein levels of tyrosinase, TRP-1, TRP-2 and MITF were also determined. Our results indicate that PCP treatment following α-MSH application significantly inhibits tyrosinase activity compared with α-MSH-stimulated cells. In addition, 1 and 1.5 mg/mL PCP treatment resulted in decreased melanin production. Melanin production in α-MSH-stimulated cells increased three-fold relative to that of vehicle-treated control cells; however, melanin content in PCP-treated cells was diminished compared with that of α-MSH-stimulated cells. The protein level of TRP-1, tyrosinase, and MITF in PCP-treated cells following α-MSH application was reduced compared with α-MSH-stimulated cells. These results suggest that PCP has the potential for skin whitening. PCP suppressed α-MSH-stimulated melanogenesis in B16F10 cells through inhibition of tyrosinase and down-regulation of TRP-1, leading to decreased melanin content. Melanin is synthesized in melanocytes and moved into keratinocytes to protect cells. Melanin biosynthesis is regulated by levels of tyrosinase, TRP-1, and TRP-2 [2,49]. Tyrosinase activity is important for controlling melanogenesis, since it functions as a catalyst of the rate-limiting reaction of the melanogenic pathway. Thus, inhibition of tyrosinase is the most common approach used for skin whitening [50,51].

We found that PCP may inhibit melanin biosynthesis through down-regulation of the p38 MAPK pathway in B16F10 cells. PCP reduced the phosphorylation of p38 MAPK, but not ERK or JNK. In addition, PCP treatment reduced the α-MSH-induced increase in MITF and decreased the phosphorylation of PKA, CREB, and GSK-β compared with α-MSH alone. This indicates that the p38 and PKA signaling pathways are associated with the effects of PCP on melanogenesis in B16F10 cells. In addition, phosphorylation of AKT was not affected, whereas GSK-3β phosphorylation was decreased in PCP + α-MSH compared with α-MSH alone. The mechanism of GSK-3β phosphorylation is not clearly understood, and further studies will be required to elucidate the role of regulating the GSK-3β level in the PCP whitening effect. Various signaling pathways regulate melanin pigment formation. Among the pathways related with the process of melanogenesis, p38 MAPK signaling was recently demonstrated to be involved in stress-induced melanogenesis [52]. Melanogenic stimuli such as α-MSH, UV irradiation, lipopolysaccharide, and placental total lipid fraction trigger a sustained increase of phospho-p38 MAPK active form [52,53,54,55]. However, the effective contribution of p38 MAPK in melanogenesis is not clearly understood. Melanogenic gene expression is regulated primarily by MITF, a transcription factor controlled by the levels of cAMP, phosphatidylinositol 3-kinase (PI3K)/AKT and MAPKs. PKA is responsible for the phosphorylation of CREB, and the activation of CREB phosphorylation induces MITF transcription. cAMP-induced PI3K inhibition results in a decrease in AKT phosphorylation and its activation [56]. Thus, AKT fails to phosphorylate GSK-3β. Subsequently, cAMP diminishes the phosphorylation of GSK-3β and promotes its activity [57]. Therefore, cAMP-mediated GSK-3β activation stimulates MITF binding to the tyrosinase promoter and lead to the activation of melanogenesis. In addition, there is much evidence that the Wnt signaling pathway is associated with MITF expression. Activation of the Wnt pathway suppresses GSK-3β activity and results in β-catenin accumulation. The accumulated β-catenin is moved into the nucleus and binds with the lymphoid-enhancing factor/T-cell factor (LEF/TCF) [58], thereby increasing MITF expression. Therefore, GSK-3β is implicated in the regulation of melanogenesis [56]. MAPKs (p38, ERK and JNK) also play essential roles in the regulation of melanogenesis [59]. It was reported that p38 activation contributes to melanin production [54] by activating CREB, which in turn activates MITF expression [60]. MITF plays an important role in melanin production by upregulating the transcription of melanogenic proteins, including tyrosinase, TRP-1 and MC1R. When p38 MAPK signaling is interrupted, CREB activation is inhibited. Consequently, the expression of melanogenic enzymes is hindered due to the limited expression of MITF. In addition, p38 MAPK was recently demonstrated to be associated with UV-induced melanogenesis, leading to the activation of MITF and increased tyrosinase expression [61]. In contrast, the ERK and JNK pathways cause down-regulation of melanin synthesis. The regulation of melanogenesis is related with activation of the MEK/ERK and PI3K/AKT signal transduction [59,62,63]. In notice, the cAMP pathway is regulated through a PI3K-dependent signal transduction cascade involving both PKA and Ras/ERK pathway in melanogenesis. In human melanoma cells, either an increase in the level of cAMP or inhibition of PI3K affects melanin formation by decreasing Akt phosphorylation [56,57].

We also examined whether the production of α-MSH-induced ROS is mediated via apoptosis signal regulating kinase 1 (ASK1), the upstream kinase of p38. We found that, following α-MSH treatment, the level of ASK-1 was enhanced slightly (data not shown); therefore, it is hypothesized that the anti-oxidative effect of PCP is not mediated by ASK1 inhibition.

## 4. Experimental Section

### 4.1. Chemicals

3-(4,5-Dimethylthiazol-2-yl)-2,5-diphenyltetrazolium bromide (MTT), 3,4-dihydroxy-l-phenylalanine (l-DOPA), arbutin, kojic acid, l-tyrosine, tyrosinase from mushrooms, phenylmethanesulfonyl fluoride (PMSF), α-melanocyte-stimulating hormone (α-MSH), 2,2-diphenyl-1-picrylhydrazyl (DPPH), 2,3-azinobis (3-ethyl-benzothiazoline-6-sulfonic acid (ABTS), synthetic melanin, l-ascorbic acid (vitamin C), and (+)-α-tocopherol (vitamin E) were purchased from Sigma-Aldrich (St. Louis, MO, USA). Primary antibodies against p-p38 MAPK (Thr180/Tyr182), p-p44/42 MAPK p-Erk 1/2 (Thr202/Tyr204), p-SAPK/JNK (Thr183/Tyr185), phospho-c-AMP-dependent kinase (PKA) (Thr197), phospho-cAMP response element-binding protein (CREB) (Ser133), phospho-glycogen kinase 3β (GSk3β) (S9), phospho-AKT (S473), and phosphor-apoptosis signal regulating kinase 1 (ASK1) (Thr845) were purchased from Cell Signaling (Danvers, MA, USA). Primary antibodies against tyrosinase-related protein 1 (TRP-1), tyrosinase-related protein 2 (TRP-2), tyrosinase, p38, ERK, JNK, GSk3β, AKT, and β-tubulin were obtained from Santa Cruz Biotechnology (Dallas, TX, USA). Primary antibodies against MITF were obtained from Abcam (Cambridge, UK). All test materials were maintained at 4 °C until use.

### 4.2. Preparation of Pomegranate Concentrate Powder

Dried PCP was obtained from Health-Love Therapeutics (Anyang, Korea), who purchased the PCP from Asya Fruit Juice and Food Industry (Ankara, Turkey). Pomegranate powder was prepared as follows. Briefly, pomegranate fruit was washed and the rind removed. Pomegranate juice and seeds were separated using a Bucher press. The juice was sterilized at high temperatures. The sterilized pomegranate juice was depectinized using pectinase. The juice was then filtered and evaporated. The concentration was diluted to 0.75–8 mg/mL with distilled water. PCP was maintained at 4 °C until use.

### 4.3. Proximate Analysis of Pomegranate Concentrate Powder

PCP was dried at 70 °C to a constant weight. Moisture content, ash, and crude fiber were determined by the Association of Official Analytical Chemists (AOAC) methods [64]. Nitrogen contents (N) of the sample were estimated, and crude protein was calculated as N × 6.25. Total lipids were extracted from the samples with chloroform/methanol (2:1, *v*/*v*) and gravimetrically quantified. The amount of total carbohydrates was obtained by determining the difference between the weight of the sample taken and the sum of its moisture, ash, total lipid, protein, and fiber content (Table 1). The PCP was found to consist of total-protein (1.79%), total-lipid (10.66%), carbohydrate (83.76%), water (3.05%), and ash (0.71%).

**Table 1:** Proximate analysis of PCP.

Component	Contents
Energy	438.14 Kcal/100 g
Carbohydrate	0.84 g/g
Sugar	0 mg/g
Total protein	0.018 g/g
Total lipid	0.11 g/g
Sodium	21.65 mg/100 g

### 4.4. HPLC Analyses of Phenolic Acids in Pomegranate Concentrate Powder

**Figure 10:**
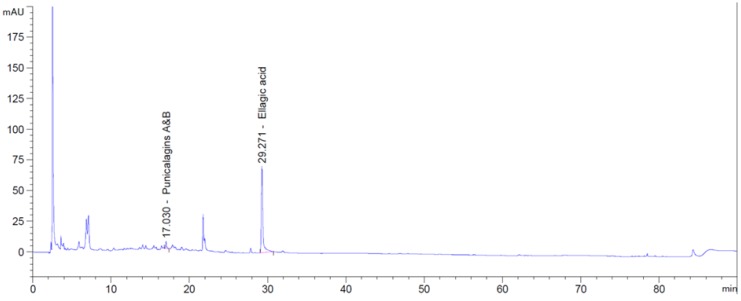
HPLC chromatogram of pomegranate concentrate powder. Peaks represent total punicalagins and ellagic acid.

One thousand mg powder was suspended in 50 mL of methanol, sonicated for 30 min, mixed, and filtered through a 0.45 μm PTFE (polytetrafluoroethylene) syringe filter. The filtrate was then analyzed by HPLC. HPLC fractionation was achieved with an Agilent Technologies HPLC 1260 System (Santa Clara, CA, USA) fitted with a Cadenza 3 μm CD-C18 column (150 × 4.6 mm) and equipped with a photodiode array detector. Samples were separated with a 0.5% acetic acid and acetonitrile elution gradient (Table 2). The column elution rate was kept at 0.7 mL/min and the injection volume was 10 μL for standard and sample. The chromatogram was taken at variant wave lengths (210 nm for Salicylic acid; 254 nm for punicalagins A and B, ellagic acid, and protocatechuic acid ethyl ester; 280 nm for gallic acid, syringic acid, *p*-coumaric acid, and cinnamic acid; 329 nm for chlorogenic acid, and *trans*-ferulic acid; 370 nm for quercetin, kaempferol, and isorhamnetin), integrated, and analyzed using an Agilent Chemstation (Agilent Technologies, Santa Clara, CA, USA), a chromatography data system. The PCP contains ellagic acid (0.36 mg/g) and punicalagins (0.07 mg/g), and the other phenolic acids were not detected (Figure 10).

**Table 2:** HPLC column elution gradient for pomegranate concentrate powder.

Time (min)	Acetic Acid (%)	Acetonitrile (%)
0	95	5
5	95	5
17	85	15
40	80	20
60	50	50
70	50	50
75	0	100
80	0	100
85	95	5
90	95	5

### 4.5. Cell Cultures

B16F10 murine melanoma cells (CRL-6475) were obtained from the American Type Cell Collection (ATCC, Manassas, VA, USA). Cells were cultured in Dulbecco’s Modified Eagle’s Medium (DMEM, Sigma-Aldrich, St. Louis, MO, USA) supplemented with 10% fetal bovine serum (FBS; Gibco, NJ, USA) and 1% penicillin (Sigma-Aldrich). Cells were cultured at 37 °C in a CO_2_ incubator with a humidified atmosphere containing 5% CO_2_. Culture medium was changed every two days.

### 4.6. Cell Viability Assay

The cell viability assay was performed using MTT [65]. B16F10 cells were incubated with various concentrations of PCP (0.75, 1, 1.5, 2, 4, and 8 mg/mL) for 72 h after pretreatment with 100 nM α-MSH for 24 h. After incubation, MTT solution was added to the wells. The insoluble derivative of MTT produced by intracellular dehydrogenase was solubilized with dimethylsulfoxide (DMSO). Absorbance of the wells was measured at 570 nm using a microplate reader (TECAN, Männedorf, Switzerland).

### 4.7. DPPH Radical Scavenging Activity Test

DPPH radical scavenging assay was performed according to the method of Impei *et al.* [66]. Briefly, 0.2 mM DPPH solution (Sigma-Aldrich) in methanol was prepared and used immediately. Each sample solution was diluted with distilled water to final PCP concentrations of 0.75, 1, 1.5, 2, 4, and 8 mg/mL, or final vitamin A and C concentrations of 1 mg/mL. Optical density (OD) was measured at 517 nm after 10 min with a UV-Vis spectrophotometer (TECAN). Free radical scavenging activity was calculated as follows: DPPH radical scavenging activity (%) = 100 − ((ODs/ODc) × 100), Where ODs represents the absorbance of the sample at 517 nm and ODc represents the absorbance of the vehicle-treated control at 517 nm. Vitamins A and C were used as positive controls.

### 4.8. ABTS Antioxidant Activity Determination

The ABTS assay was performed using the method of El-Said *et al.* [42] with some modifications. Stock solutions of 7.4 mM ABTS^+^ and 2.6 mM potassium persulfate were prepared. Next, a working solution was made by mixing each stock solution and reacting solution for 12 h at room temperature in the dark. The solution was then diluted by mixing 1 mL ABTS^+^ solution with 30 mL methanol to obtain an absorbance of 0.70 ± 0.02 units at 734 nm using a spectrophotometer. Fresh ABTS^+^ solution was prepared for each assay. PCP (150 mL) was allowed to react with 2850 mL ABTS^+^ solution for 2 h in the dark. Absorbance was then determined at 734 nm using a spectrophotometer. Additional dilutions were prepared if the measured ABTS value exceeded the linear range of the standard curve. Antioxidant activity was calculated as follows: ABTS scavenging activity (%) = 100 − ((ODs/ODc) × 100).

### 4.9. Total RNA Extraction and RT-PCR Analysis

Total RNA was extracted from liver tissue samples using TRIzol reagent (Invitrogen, Carlsbad, CA, USA). cDNA was synthesized via a reverse transcriptase (RT) reaction, and polymerase chain reaction (PCR) amplification was performed using a thermal cycler (Bio-Rad, Hercules, CA, USA). Real-time RT-PCR analysis was carried out with a Bio-Rad CFX96TM (Bio-Rad) using iTaq™ SYBR Green SuperMix (Bio-Rad). Primers were obtained from Bioneer (Daejeon, Korea). Primer sequences for mouse genes are as follows: modulatory subunit of GCL (GCLM), 5′-AGGAGCTTCGGGACTGTATT-3′ and 5′-TGGGCTTCAATGTCAGGGAT-3′; catalytic subunit of GCL (GCLC), 5′-CAAGGACGTGCTCAAGTGG-3′ and 5′-GTAACTCCCATACTCTGGTCTC-3′; superoxide dismutase 1 (SOD-1), 5′-GTTCCACGTCCATCAGTATG-3′ and 5′-ACACGATCTTCAATGGACAC-3′; glutathione peroxidase 1 (GPx-1), 5′-GGGACTACACCGAGATGAAC-3′ and 5′-TCACTTCGCACTTCTCAAAC-3′; glutathione reductase (GSR), 5′-CAGGCATGATAAGGTACTGAG-3′ and 5′-CATCTGGAATCATGGTCGTG-3′; catalase (CAT), 5′-CAAAGGTGTTGAACGAGGAG-3′ and 5′-TGTAGGTGTGAATTGCGTTC-3′, and hypoxanthine-guanine phosphoribosyl transferase (HPRT), 5′-AGATGTCATGAAGGAGATGG-3′ and 5′-TACAGTAGCTCTTCAGTCTG-3′.

### 4.10. Tyrosinase Assay

Tyrosinase activity was determined according to the method of Masamoto [67]. Briefly, aliquots (0.05 mL) of PCP (0.75, 1, 1.5, 2, 4, and 8 mg/mL) were mixed with 0.5 mL l-DOPA (Sigma-Aldrich) solution (1.25 mM) and sodium acetate buffer solution (0.05 M, pH 6.8), and preincubated at 25 °C for 10 min. An aqueous solution of mushroom tyrosinase (110 U/mL; Sigma-Aldrich) was then added to the mixture. This solution was immediately monitored for the formation of dopachrome by measuring the linear increase in OD at 475 nm with a UV-Vis spectrophotometer, and tyrosinase inhibitory activity was calculated using the following equation: tyrosinase inhibitory activity (%) = 100 − ((ODs/ODc) × 100), where ODs represents the absorbance of the experimental sample at 475 nm and ODc represents the absorbance of the vehicle-treated control at 475 nm. Results are reported as the IC_50_ (the concentration at which the percentage inhibition of tyrosinase activity was 50%). Kojic acid (25, 50, 100, 200, and 400 μM) was used as a standard under the same experimental conditions.

### 4.11. Cellular Tyrosinase Assay

Cellular tyrosinase activity was determined using a previously described method [68]. B16F10 cells were incubated with various concentrations of PCP (0.75, 1, and 1.5 mg/mL) and kojic acid (50, 100, 200, and 400 μM) for 72 h after pretreatment with 100 nM α-MSH for 24 h. At the end of this treatment, cells were lysed with phosphate buffer (pH 6.8) containing 1 mM PMSF. Lysates were clarified by 10 min centrifugation at 13,000 rpm. The amount of protein in each lysate was quantified and concentration adjusted with lysis buffer. Next, 40 μL of each lysate was mixed with 200 μL 10 mM l-DOPA and incubated for 30 min at 37 °C. This solution was immediately monitored for the formation of dopachrome by measuring the linear increase in OD at 475 nm with a UV-Vis spectrophotometer, and tyrosinase activity was calculated using the following equation: tyrosinase activity (%) = ((ODs/ODc) × 100), where ODs represents the absorbance of the experimental sample at 475 nm and ODc represents the absorbance of the vehicle-treated control at 475 nm. Kojic acid (200 μM) was used as a standard under the same experimental conditions.

### 4.12. Melanin Formation Test in B16/F10 Melanoma Cells

Melanin content was measured using a method described previously [59] with slight modifications. Cells were exposed to various concentrations of PCP (0.75, 1 or 1.5 mg/mL) and arbutin (0.5, 1, 2, and 4 mM) for 72 h, and then in the presence or absence of 100 nM α-MSH (Sigma-Aldrich) for 24 h. The number of cells in each sample was counted, and the same total number of cells was obtained. Following treatment, cells were washed with phosphate-buffered saline (PBS) and dissolved in 1 M NaOH containing 10% DMSO for 1 h at 90 °C. Absorbance was measured at 400 nm using a microplate reader. Total melanin in each cell suspension was determined by recording the absorbance of each suspension at 405 nm. Melanin content was calculated by interpolating the results from a standard curve generated using the absorbance of known concentrations of synthetic melanin (Sigma-Aldrich). Arbutin (2 mg/mL) was used as a standard under the same experimental conditions.

### 4.13. Western Blot Analysis

Cells were dissolved in RIPA buffer (50 mM Tris, pH 7.4, 150 mM NaCl, 1 mM EDTA, 1% NP40) containing a protease inhibitor cocktail (Sigma-Aldrich). Protein concentration was determined using a DC Protein Assay Kit (Bio-Rad). Protein samples were loaded onto 10% sodium dodecyl sulfate (SDS)-polyacrylamide gels and separated according to molecular weight. Gels were transferred to polyvinylidene fluoride (PVDF) membranes (Whatman, Dassel, Germany) using a Trans-Blot^®^ Semi-Dry Cell (Bio-Rad). Membranes were blocked with 5% skim milk or 5% bovine serum albumin (BSA) for 1 h. Primary antibodies (p-ERK, p-p38, p-JNK, TRP-1, TRP-2, tyrosinase, or β-tubulin) were added and incubated overnight at 4 °C. Membranes were then incubated with secondary antibodies for 1 h. Enhanced chemiluminescence (ECL) solution was added and the membranes were exposed to film (Kodak BioMax XAR film, Kodak, Japan).

### 4.14. Statistical Analyses

All data are expressed as the mean ± standard deviation (SD) from three independent experiments. Multiple comparison tests for different dosage groups were conducted. The homogeneity of variance was examined using Levene’s test. If this test indicated no significant deviation from the variance of homogeneity, the obtained data were analyzed using one-way analysis of variance (ANOVA) followed by the least-significant differences (LSD) multiple-comparison test to determine which pairs in the group were significantly different. If Levene’s test showed significant deviation from variance homogeneity, the non-parametric Kruskal–Wallis *H* test was conducted. When a significant difference was observed with the Kruskal–Wallis *H* test, the Mann-Whitney *U-*test was used to identify specific pairs in the group that were significantly different. The IC_50_ value for each *in vitro* assay was calculated using probit methods. Statistical analyses were conducted using SPSS for Windows (release 14.0K; SPSS, Chicago, IL, USA).

## 5. Conclusions

Collectively, these results suggest that PCP exerts whitening effects. PCP effectively decreased tyrosinase activity and melanin production in B16F10 cells, which may be attributed to its inhibitory action on melanogenesis via inactivation of the p38 signaling pathway.

Our results indicate that PCP has potential antioxidant characteristics and exerts strong DPPH radical scavenging effects. PCP increases transcript levels of GPx-1. In addition, PCP exerts whitening effects by effectively decreasing tyrosinase activity and melanin production in B16F10 cells, which is attributed to its inhibitory action on melanogenesis via inactivation of the p38 and PKA/CREB signaling pathway.
